# Foraging Ranges of Immature African White-Backed Vultures (*Gyps africanus*) and Their Use of Protected Areas in Southern Africa

**DOI:** 10.1371/journal.pone.0052813

**Published:** 2013-01-30

**Authors:** W. Louis Phipps, Stephen G. Willis, Kerri Wolter, Vinny Naidoo

**Affiliations:** 1 Department of Paraclinical Sciences, University of Pretoria, Onderstepoort, Guateng, South Africa; 2 School of Biological and Biomedical Sciences, Durham University, Durham, Co. Durham,United Kingdom; 3 Vulture Programme, Plot 121 Rietfontein, Brits District, North West Province, South Africa; 4 Biomedical Research Centre, University of Pretoria, Onderstepoort, Guateng, South Africa; University of Marburg, Germany

## Abstract

Vultures in the *Gyps* genus are declining globally. Multiple threats related to human activity have caused widespread declines of vulture populations in Africa, especially outside protected areas. Addressing such threats requires the estimation of foraging ranges yet such estimates are lacking, even for widespread (but declining) species such as the African white-backed vulture (*Gyps africanus*). We tracked six immature African white-backed vultures in South Africa using GPS-GSM units to study their movement patterns, their use of protected areas and the time they spent in the vicinity of supplementary feeding sites. All individuals foraged widely; their combined foraging ranges extended into six countries in southern Africa (mean (± SE) minimum convex polygon area  = 269,103±197,187 km^2^) and three of the vultures travelled more than 900 km from the capture site. All six vultures spent the majority of their tracking periods outside protected areas. South African protected areas were very rarely visited whereas protected areas in northern Botswana and Zimbabwe were used more frequently. Two of the vultures visited supplementary feeding sites regularly, with consequent reduced ranging behaviour, suggesting that individuals could alter their foraging behaviour in response to such sites. We show that immature African white-backed vultures are capable of travelling throughout southern Africa, yet use protected areas to only a limited extent, making them susceptible to the full range of threats in the region. The standard approach of designating protected areas to conserve species is unlikely to ensure the protection of such wide-ranging species against threats in the wider landscape.

## Introduction

Vultures in the *Gyps* genus are obligate scavengers and are the main consumers of ungulate carcasses in African savannahs [1], [2]. Their energy efficient soaring flight, keen eyesight and social foraging behaviour enable them to locate sparsely and unpredictably distributed carcasses over a large area, often before their mammalian competitors [3], [4]. Their dependence upon such a transient and seasonally variable food supply results in high levels of competition among large gatherings of feeding vultures as they attempt to secure a meal whenever an opportunity arises [4]. The ability of *Gyps* vultures to rapidly locate and consume the soft tissues of dead ungulates provides important ecosystem services by recycling carcasses, keeping energy flows high in food webs, and limiting the development and spread of disease [5].

All eight *Gyps* vulture species found globally are currently declining due to multiple threats including habitat loss, reduced food availability, direct persecution, and emerging threats such as climate change and fatal collisions with wind turbines and electricity cables [6]–[8]. Their delayed maturity (African white-backed vultures (*G. africanus*) generally breed after their fourth year [4]) and relatively low reproductive rates make vulture populations especially vulnerable to high mortality rates [7]. Since the 1990s three species of *Gyps* vultures have declined by more than 95% in parts of Asia mainly due to accidental poisoning after consuming carcasses of domestic livestock previously treated with the veterinary non-steroidal anti-inflammatory drug (NSAID), diclofenac [9], [10]. This rapid collapse of Asian vulture populations has resulted in changes to scavenger community composition and a consequent increase in the incidence of diseases such as rabies and anthrax in humans [7]. African *Gyps* vultures are equally sensitive to the toxic effects of diclofenac and other NSAIDs, raising concerns of potential rapid population declines in the future [11], [12].

Large declines in vulture populations have been documented in many parts of Africa, especially outside protected areas [13]–[15]. Two of the most serious threats to African vultures are food shortages caused by improved animal husbandry and over-harvesting of wild ungulate populations, and mass poisoning of vultures when they consume carcasses laced with poisons intended to kill predators of livestock [4], [13]. For example, increasingly frequent poisoning incidents are the most likely cause of a 52% decline in *Gyps* vulture numbers in the Masai Mara ecosystem in Kenya over a 30 year period [13]. Their gregarious feeding behaviour and ability to forage over large areas make *Gyps* vultures particularly susceptible to mass poisoning events which tend to occur most frequently on unprotected farmland [4].

Additional threats to vultures in Africa include fatal collisions and electrocutions with power lines, illegal harvesting for the traditional beliefs market, and the disturbance or loss of breeding sites, all of which are more prevalent in unprotected areas [7], [16]. Consequently, several studies have found that vultures are becomingly increasingly restricted to protected areas in different regions of Africa and the importance of protecting them beyond the boundaries of wildlife reserves is considered paramount to their future conservation [7], [13]–[15]. In an effort to provide an uncontaminated source of supplementary food for vultures outside protected areas “vulture restaurants” have been used in southern Africa since the latter half of the twentieth century [17]. Although vulture survival rates have increased in some areas with supplementary feeding schemes [17], the impact of supplementary feeding on vulture foraging ecology is not fully understood [18].

The African white-backed vulture is widespread in sub-Saharan Africa and is often the most numerous vulture species in its typical habitat of lowland open wooded savannah where it nests in trees in loose colonies [4]. They forage in groups and form extensive social foraging networks by soaring on and gliding between thermal air currents which they rely upon to become airborne and gain altitude due to their large body size [2], [3]. Gatherings of large numbers of individuals competing at a carcass are typical and give rise to voracious feeding activity, with an individual vulture able to fill its crop with 1 kg of soft tissue in 2 minutes [1], [4]. Although its global population has been estimated at 270,000 individuals the species has suffered significant declines throughout its range, prompting the recent upgrading of its conservation status from Near Threatened to Endangered on the IUCN Red List [16]. Through re-sightings of marked individuals in southern Africa, immature African white-backed vultures are known to travel extensively [19], but a greater understanding of their movement patterns, foraging ecology and use of protected areas is required to assess their susceptibility to different threats [13].

In this study we use GPS telemetry to study the movement patterns of six immature African white-backed vultures caught from the wild in South Africa. We use three widely applied range estimation methods to determine the size, extent and seasonal variation of the vultures' foraging ranges. We also assess their utilization of protected areas and supplementary feeding sites. Although the survival of breeding adult vultures is essential for the persistence of their populations we prioritised tracking immature birds as we expected them to range further and consequently be most exposed to multiple threats across the wider landscape during this phase of their life [4], [17], [20]. As immature birds can comprise >50% of the population of African white-backed vultures [21] any impacts on this cohort will have a large impact on the total population size, even if adults, and therefore productivity, are relatively unaffected in the short term. The relative impact of increased immature mortality due to human-induced versus natural mortality is poorly understood. This study therefore provides a first insight into this endangered species' foraging patterns in southern Africa based on continuous GPS tracking data for the first time.

## Methods

### Vulture captures

**Figure 1:**
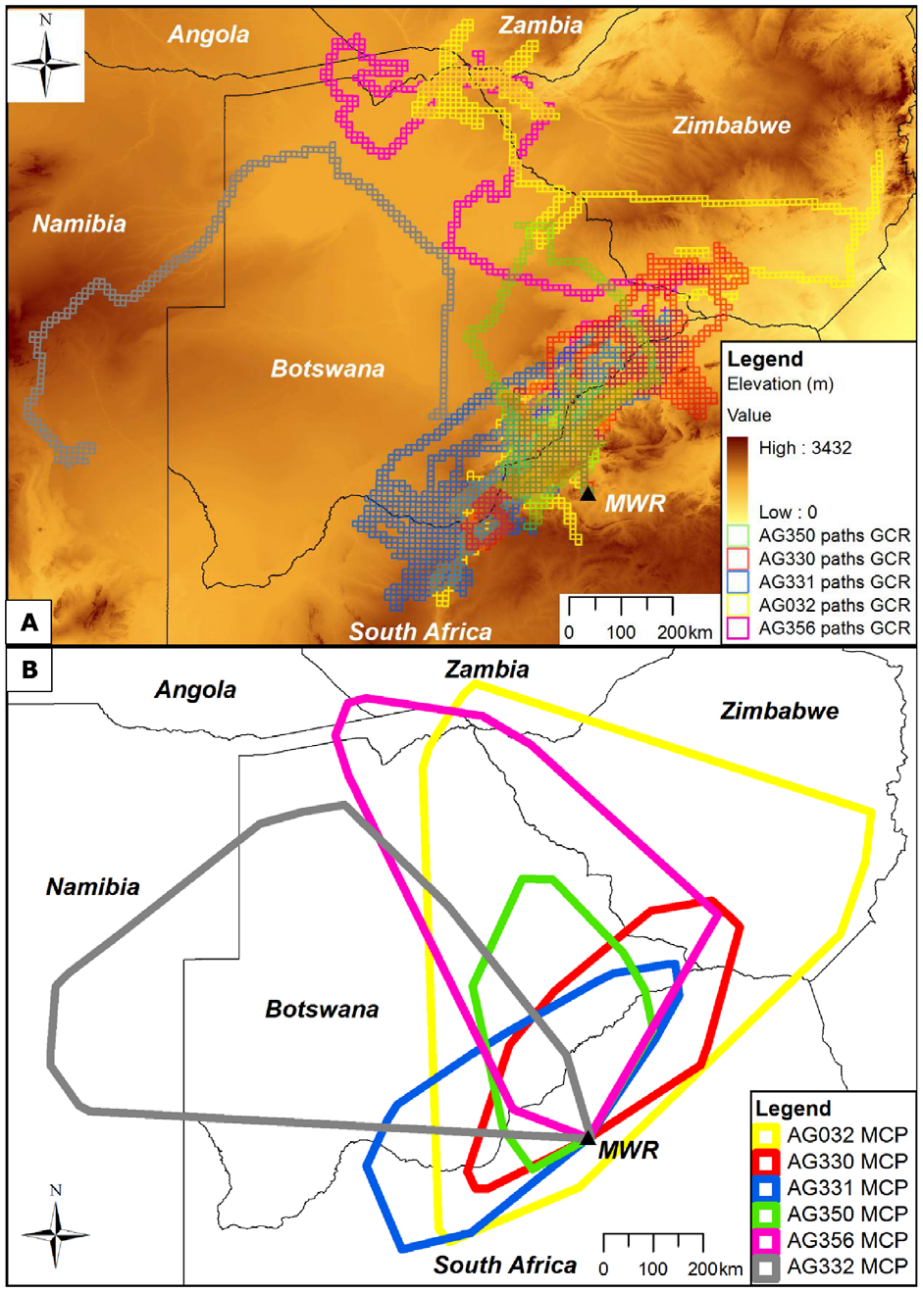
Foraging ranges represented by (A) path GCRs and (B) MCPs for six immature African white-backed vultures. Path GCRs (A) represent 10×10 km grid cells intersected by a continuous line between all consecutive GPS locations recorded during the total tracking period of each vulture. MCPs (B) were created by connecting the outermost GPS locations recorded for each vulture. Mankwe Wildlife Reserve capture site is indicated by a black triangle and “MWR”.

Vultures were caught at a supplementary feeding site for mammalian and avian scavengers at Mankwe Wildlife Reserve (MWR; 25°13′S, 27°18′E), approximately 4 km east of Pilanesberg National Park (25°14′S, 27°05′E) in the North West Province of South Africa (Fig. 1). A walk-in cage trap (6×3×3 m) constructed from a lightweight steel frame overlaid with wire mesh and baited with domestic livestock or wild ungulate carcasses was used to catch the vultures [22]. Six immature African white-backed vultures were caught and fitted with GPS-GSM tracking units during three separate captures.

### GPS-GSM tracking units

Hawk105 GPS-GSM tracking units (Africa Wildlife Tracking Ltd., Pretoria, South Africa; www.awt.co.za) were secured onto the back of each vulture using a Teflon^®^ ribbon backpack-style harness enclosed in flexible plastic tubing to prevent skin abrasions [23]. Each unit weighed 170 g (*c.* 3.1% of the mean mass of an African white-backed vulture [4]) and was encased in hardened epoxy resin for protection and waterproofing. The units were set to record GPS locations (∼10 m accuracy), altitude above sea level, speed and direction of travel, date, time and temperature at three times per day: 07:00, 11:00 and 15:00. The tracking units also recorded a positional dilution of precision (PDOP) value as a measure of the accuracy of each GPS location [24]. The data were transmitted daily by SMS to a secure online database via the GSM network. Whenever a vulture was in an area without GSM coverage, up to 20,000 data points could be stored on the unit which were then transmitted when it returned to an area with coverage. It was anticipated that each unit would record and transmit data for approximately one year. Yellow patagial tags inscribed with a unique four character code were also attached through the patagia of both wings of each captured vulture to allow visual identification of individuals after release.

The procedures were approved by the Animal Use and Care Committee of the University of Pretoria (Protocol: V033-09). Permits for the capture and handling of vultures and the fitting of tracking units were granted by the Department of Agriculture, Conservation, Environment and Rural Development, North West Provincial Government, Republic of South Africa (Permit: 000085 NW-09).

### Data analysis

For all spatial analyses the GPS locations were projected to the UTM coordinate system (WGS 1984 UTM Zone 35S). The degree of autocorrelation of each individual's GPS locations was assessed using Schoener's [25] index of autocorrelation in Home Range Tools extension [26] for ArcGIS®.

Distances between consecutive GPS locations were calculated for each vulture. A very conservative estimation of the total distance travelled per day by an individual was obtained by summing the distances between all GPS locations recorded in a 24 hour period (i.e. (07:00–11:00) + (11:00–15:00) + (15:00–07:00)). For each vulture, the total distance travelled, the mean distance between consecutive locations, and the mean and maximum distance travelled per day were calculated.

Estimates of the foraging ranges traversed by each vulture during their total tracking periods were calculated using three methods to account for potential variation among techniques [27]. Firstly, foraging ranges were delineated with Minimum Convex Polygons (MCPs) using all recorded GPS locations [28]. Although MCPs have a tendency to overestimate the actual area occupied by an animal by including outlying locations [28], they were used here to compare our estimates with previous tracking studies on *Gyps* vultures (e.g. [20]). Incremental area analysis was carried out in Ranges7 [29] to investigate whether the size of the vultures' foraging ranges represented by MCPs reached an asymptote during the total tracking period [28]. For each individual, MCPs were created by sequentially adding consecutive locations until all locations were used to produce the MCP for the total tracking period. A foraging range area curve was then plotted and asymptotes were identified visually [28].

Secondly, fixed kernel density estimation (KDE) was used to delineate 95% and 50% contours to represent the overall and core foraging ranges, respectively [30]. An *ad hoc* bandwidth (*h_ad hoc_*) designed to reduce over-smoothing of the KDE contours [27] was used for KDE calculations. The value of *h_ad hoc_* was determined by reducing the reference bandwidth (*h_ref_*) in increments of 0.05 until the 95% contour became contiguous with no lacunae (i.e. *h_ad hoc_*  = 0.95× *h_ref_*, 0.90× *h_ref_*, 0.85× *h_ref_*, etc.; [40], [41]). A 1000×1000 m raster cell size was used for KDE calculations. The Home Range Tools extension [26] for ArcGIS® was used for MCP and KDE analysis.

Thirdly, grid cell range (GCR) estimates [28] were calculated using Hawth's Analysis Tools v3.27 [31]. A 10×10 km grid was intersected by the continuous line connecting all consecutive locations for each individual, which represented the shortest assumed path travelled between consecutive locations. Summing the area of the grid cells that were intersected by the path linking the consecutive locations provided an estimate of the size of the overall foraging range, termed the path GCR [32]. The number of GPS locations in each grid cell was counted and core areas (core GCRs) were identified as the cells in which the number of locations was greater than the mean number per cell across the overall range [33]. Path GCR estimates were also calculated for separate complete months (i.e. months with data on >90% of days) for each vulture in order to identify any seasonal patterns in ranging behaviour.

Vulture utilisation of officially protected areas was investigated separately for each vulture at the foraging range scale based on use-availability analysis [34]. A polygon shapefile of protected areas in southern Africa was created using data from the 2010 World Database on Protected Areas (WDPA) containing all IUCN category I-VI protected areas [35] and ‘national other areas’ (i.e. protected areas uncategorized by IUCN) polygons from the 2003 WDPA [36]. The two datasets were merged into a single polygon shapefile. All areas outside the protected areas polygons were designated as unprotected areas.

Ivlev's electivity index [37] was used to evaluate whether protected areas were used by each vulture in proportion to their availability, and was calculated as *E_i_*  =  (*U_i_ – A_i_*)/(*U_i_ + A_i_*), where *E_i_* is the electivity index value, and *U_i_* and *A_i_* are the use and availability of protected areas, respectively. The proportion of each vulture's 95% KDE contour occupied by protected areas defined their availability to each vulture. Use of protected areas was defined as the proportion of stationary (i.e. <10 km**·**h^−1^) GPS locations that were recorded inside protected areas within the 95% KDE contour. We also calculated the proportion of each vulture's 50% KDE contours occupied by protected areas to estimate their use at the core foraging range scale. Ivlev's electivity index ranges from −1 (completely avoided) to +1 (maximum positive selection), with zero indicating that use of protected areas was proportional to their availability, while positive and negative values indicate greater and less use of protected areas than expected, respectively.

To estimate use of supplementary feeding sites, the proportion of stationary GPS locations recorded within 1 km of known supplementary feeding sites for scavengers in southern Africa was calculated separately for each vulture. The supplementary feeding sites were identified from a combination of databases compiled during questionnaire surveys between 2000 and 2010 (K. Wolter, unpublished data). Analyses were conducted for the total tracking periods and separately for each complete month for all vultures.

## Results

The six vultures fitted with tracking units were all less than four years of age and all but one were tracked continuously for at least 200 days. The sixth vulture (AG332) was tracked for 101 days before the tracking unit stopped transmitting data. The six tracking units recorded a mean of 99.44±0.25% of expected GPS locations, with a high mean (± SE) accuracy of 2.4±0.02 PDOP (n = 4,326 locations).

### Foraging ranges and distances travelled

**Figure 2:**
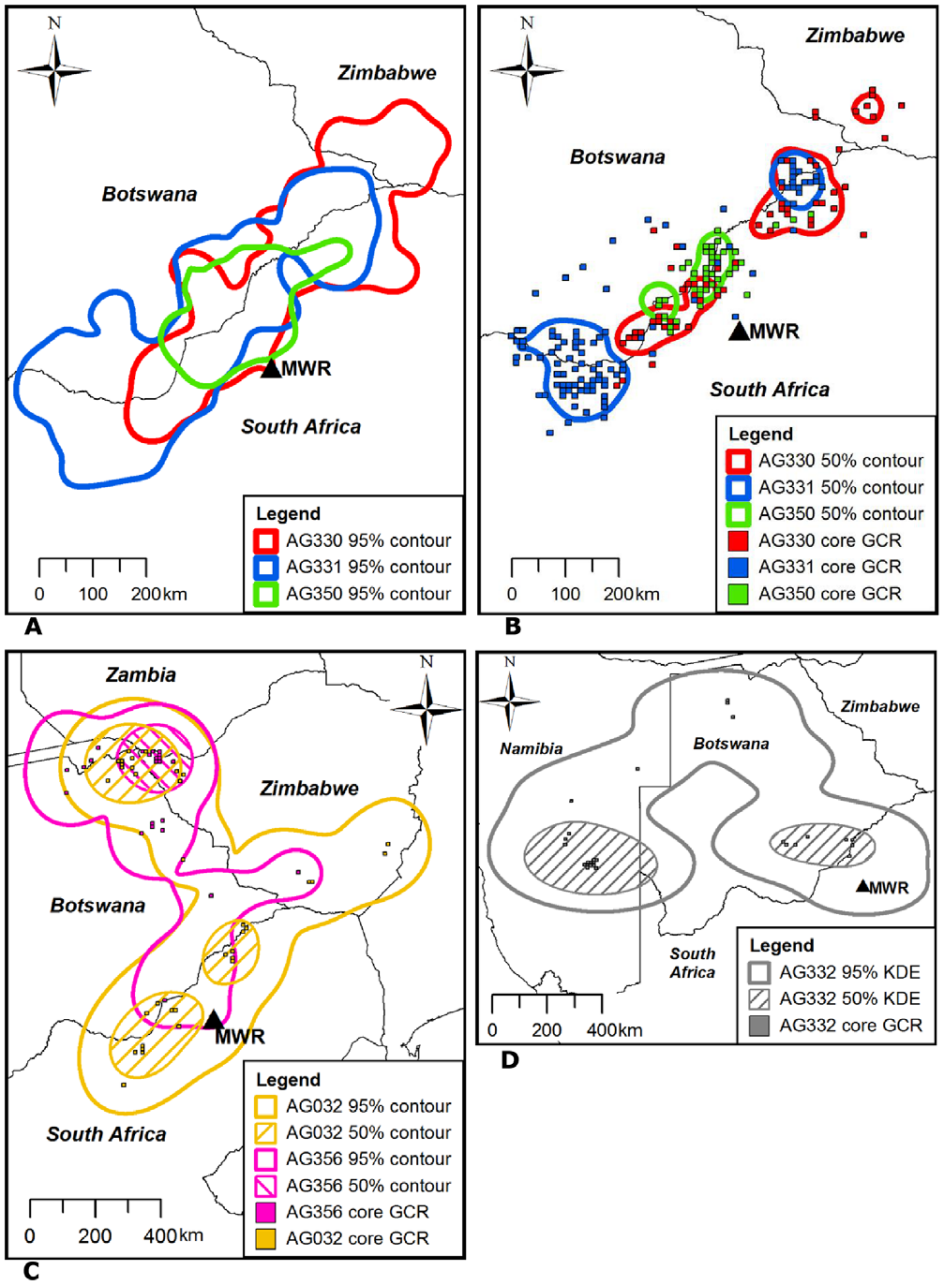
Overall and core foraging ranges for each individual. 95% KDE contours represent overall foraging ranges, 50% KDE contours and core GCRs represent core foraging ranges. (A) and (B) show the foraging ranges for AG330, AG331 and AG350; (C) shows the foraging ranges for AG032 and AG356; (D) shows the foraging ranges for AG332. Mankwe Wildlife Reserve capture site is indicated by a black triangle and “MWR”.

The combined foraging ranges of all six vultures extended across extensive areas of southern Africa (Fig. 1). The vultures traversed path GCRs covering an average (± SE) of 56,683±9,210 km^2^ (Table 1, Fig. 1A). MCPs included large areas that were never visited by the vultures (Fig. 1B). KDE contours delineated realistic range boundaries for three vultures (AG330, AG331 and AG350; Fig. 2A, B), but for the three widest ranging vultures they incorporated areas that were never visited (Fig. 2C, D). Hereafter, unless otherwise stated, foraging range areas presented in the text are from GCR estimates which provided the most realistic, but conservative, representation of the vultures' actual movements.

**Table 1:** Foraging range and distance estimates for six immature African white-backed vultures.

			Foraging range area estimates (km^2^)					Distance estimates (km)			
Vulture ID	Tracking period (days)	GPS locations	100% MCP	95% KDE	50% KDE	Path GCR	Core GCR	Total distance travelled	Mean (± SE) distance between consecutive locations	Maximum distance travelled/day	Mean (± SE) total distance travelled/day
**AG330**	301	896	144,568	125,861	29,714	59,400	5,700	7,699.03	8.60±0.61	183.90	25.84±2.04
**AG331**	313	935	155,301	145,854	28,044	89,800	11,400	15,293.49	16.37±0.83	267.43	48.86±2.59
**AG350**	300	898	124,492	47,132	8,825	43,100	4,200	11,273.75	12.57±0.67	241.63	37.58±2.23
**AG356**	226	680	332,451	342,413	34,967	44,900	2,300	5,032.54	7.41±0.62	171.22	22.27±2.13
**AG032**	206	616	588,705	582,795	122,465	74,500	4,000	8,453.76	13.75±1.01	223.48	41.04±2.95
**AG332**	101	301	439,520	765,483	132,662	28,400	2,700	2,502.17	8.34±1.14	160.17	24.77±3.94

MCPs including all recorded GPS locations (100% MCP), 95% contours from kernel density estimation (KDE), and path grid cell ranges (GCRs) represent overall foraging ranges. 50% KDE contours and core GCRs represent core foraging ranges. Total distances travelled during total tracking periods, mean (± SE) distances between consecutive GPS locations, maximum and mean (± SE) total distances travelled per day are shown for each vulture. The tracking period and number of GPS locations recorded are also shown.

Foraging range area curves from incremental area analysis reached asymptotes that lasted for at least 50 days for all vultures apart from AG332 (Fig. S1). A general pattern of settled periods followed by exploratory movements beyond the existing MCP boundary occurred for all vultures. For all vultures foraging range area curves were asymptotic at the end of their tracking periods, indicating that the tracking periods were sufficient to provide representative range estimates. The GPS location datasets for each of the six vultures were individually significantly autocorrelated, with a mean (± SE) Schoener's index value of 0.019±0.011.

The mean (± SE) and maximum speed of all recorded moving (≥ 10 km·h^−1^) GPS locations was 51.13±0.59 km·h^−1^ and 107 km·h^−1^ (n = 747), respectively. The mean (± SE) distance travelled per day ranged from 22.27±2.13 km for AG356 to 48.86±2.59 km for AG331 (Table 1). Three vultures travelled more than 220 km in a single day. AG331 travelled the furthest during the total tracking period, moving 15,293 km in 313 days. GPS locations were recorded more than 900 km from the capture site for three vultures. Following its capture, AG332 travelled north through Botswana before proceeding to south-east Namibia, travelling 2,502 km and covering an overall foraging range of 28,400 km^2^ in 101 days (Fig. 2D). AG356 also travelled north immediately after capture, moving through eastern Botswana and western Zimbabwe to the Victoria Falls region (17°55′S, 25°50′E) of Zimbabwe where it remained for a three month period (Fig. 2C) before travelling through the Caprivi Strip (Namibia) to south-west Angola, returning to north-east Zimbabwe through northern Botswana. After spending 3.5 months in the North West and Limpopo Provinces of South Africa, AG032 travelled north through southern Zimbabwe to north-east Botswana and north-west Zimbabwe. During the total tracking period of 206 days AG032 travelled over 8,454 km and occupied an overall range of 74,500 km^2^, at one point moving 520 km across the width of south-central Zimbabwe in 2.5 days. AG032 and AG356 entered a total of five and six different countries, respectively (Fig. 2C).

The foraging ranges of the remaining three vultures (AG330, AG331 and AG350) extended across the Botswana-South Africa and Zimbabwe-South Africa borders, orientated in a south-west to north-east direction from the Vryburg (21°03′S, 29°21′E) region of South Africa to the West Nicholson (26°57′S, 24°43′E) area of south-west Zimbabwe (Fig. 2A). KDE and GCR analyses showed that these three vultures, as well as AG032, used at least two core foraging areas bisected by the South Africa-Botswana border (Fig. 2B).

**Figure 3:**
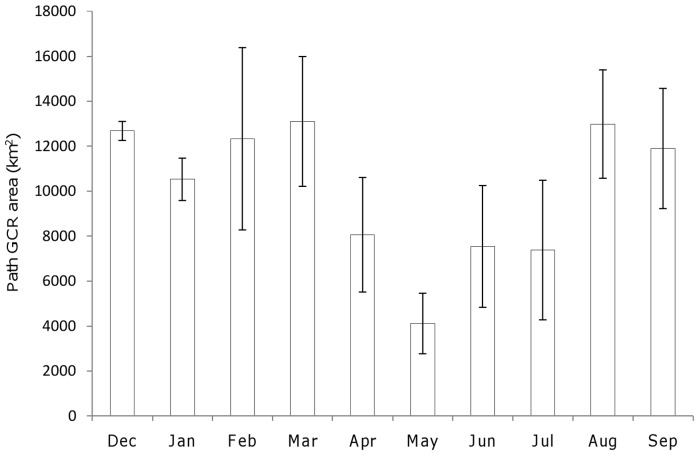
Mean (± SE) path GCR estimates for individual months for six immature African white-backed vultures. Due to differences in tracking periods for individuals, estimates were calculated for four vultures for December to March, and five vultures from April to September, inclusive.

Monthly path GCR estimates ranged from 600 to 22,200 km^2^ (mean ± SE  = 9,878±846 km^2^; n = 46 months). For three out of five vultures the smallest path GCR estimates were recorded in May (Fig. 3). The five vultures that were tracked during both the wet summer (December to April) and dry winter (May to September) periods occupied significantly larger average monthly path GCRs during summer months (mean ± SE  = 12,162±1,217 km^2^; n = 5 vultures) compared to winter months (mean ± SE  = 8,874±1,720 km^2^; n = 5 vultures) (Wilcoxon signed-rank test: *Z* = −2.20, *p* = 0.043).

### Utilisation of protected areas

Protected areas occupied a mean (± SE) of 4.33±1.50% of the 95% KDE contours of the three vultures that spent the majority of their tracking periods either side of the South Africa-Botswana border (AG330, AG331 and AG350), compared to 32.22±9.75% of the 95% KDE contours of the two vultures that travelled to northern Botswana and Zimbabwe (AG032 and AG356; Table 2). A mean (± SE) of 5.21±0.88% of stationary GPS locations within the 95% KDE contours of AG330, AG331 and AG350 were recorded inside protected areas, compared to 35.30±1.13% for AG356 and AG032 (Table 2). Protected areas occupied a mean (±SE) of 3.15±1.58% of the 50% KDE contours of AG330, AG331 and AG350, compared to 38.62±11.63% for AG356 and AG032 (Table 2).

**Table 2:** Availability and use of protected areas by six immature African white-backed vultures at the overall and core foraging range scales.

Vulture ID	Land use	Availability in 95% KDE (%)	Use at overall foraging range scale (%)	Use at core foraging range scale (%)
AG330	PA	7.20	6.90	4.63
	Non-PA	92.80	93.10	95.37
AG331	PA	2.12	3.97	0.00
	Non-PA	97.88	96.03	100.00
AG350	PA	3.66	4.76	4.82
	Non-PA	96.34	95.24	95.18
AG356	PA	41.97	34.16	50.25
	Non-PA	58.03	65.84	49.75
AG032	PA	22.47	36.43	26.99
	Non-PA	77.53	63.57	73.01
AG332	PA	27.56	8.72	4.66
	Non-Pa	72.44	91.28	95.34

The proportion of each vulture's 95% KDE contour occupied by protected areas defined their availability to each vulture. At the overall foraging range scale use of protected areas was defined as the proportion of stationary (i.e. <10 km·h^−1^) GPS locations within the 95% KDE contour that were recorded inside protected areas. The proportion of each vulture's 50% KDE contours occupied by protected areas defined their use at the core foraging range scale.

At the overall foraging range scale Ivlev's electivity index values (Fig. S2A) indicated that more stationary GPS locations were recorded inside protected areas than expected for three vultures, while fewer than expected were recorded inside protected areas for the other two. At the core foraging range scale Ivlev's electivity index values (Fig. S2B) indicated that protected areas occupied a similar proportion of the 50% KDE contours to the 95% KDE contours for three of the vultures, but a smaller proportion of the 50% KDE contours than expected for the other two. Protected areas were completely absent from the 50% KDE contours of AG331, resulting in an Ivlev's electivity index value indicating maximum avoidance.

**Figure 4:**
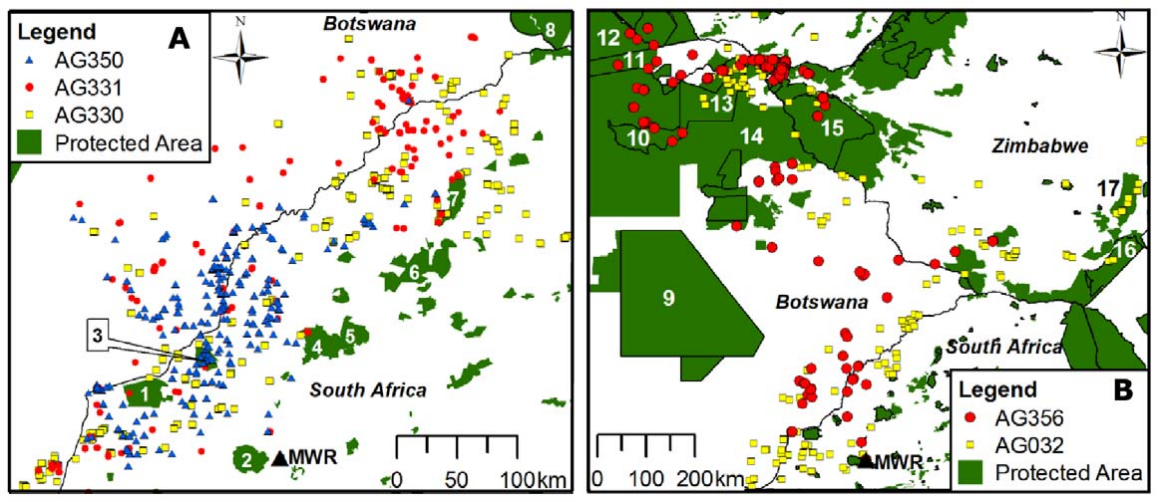
Stationary GPS locations of immature African white-backed vultures in relation to protected areas. (A) shows stationary GPS locations from AG330, AG331 and AG350 in relation to protected areas in the North West and Limpopo Provinces of South Africa: 1 =  Madikwe GR; 2 =  Pilanesberg NP; 3 =  Atherstone NR; 4 =  Marakele NP; 5 =  Welgevonden NR; 6 =  Lapalala, Moepel *et al.* reserves; 7 =  Wonderkop NR; 8 =  Tuli conservation area. (B) shows stationary GPS locations from AG356 and AG032 in relation to protected areas across southern Africa: 9 =  Central Kalahari NP; 10 =  Moremi GR; 11 =  Caprivi GR; 12 =  Luiana NP (Angola); 13 =  Chobe NP; 14 =  Wildlife Management Areas; 15 =  Hwange NP; 16 =  Gonarezhou NP; 17 =  Save Conservancy. Protected area data are from WDPA [32], [33].

South African protected areas were not visited regularly by any vultures (Fig. 4A), with AG032 never entering a South African protected area in a period of more than 3 months. Pilanesberg NP (25°14′S, 27°05′E) and other relatively large conservation areas in the North West and Limpopo Provinces of South Africa were never visited by any of the vultures, while only two and five stationary locations (both from AG331) were recorded inside Madikwe Game Reserve (24°45′S, 26°14′E) and Marakele NP (24°24′S, 27°35′E) respectively. None of the three vultures that spent the majority of their tracking periods in South Africa or southern Botswana spent extended periods inside protected areas.

The two vultures that travelled more extensively through southern Africa visited protected areas more regularly (Fig. 4B), particularly in northern Botswana and Zimbabwe in the Kavango-Zambezi Transfrontier Conservation Area (TFCA), where they spent extended periods inside wildlife reserves such as Chobe NP (18°08′S, 24°43′E) and associated wildlife management areas (WMAs) in northern Botswana, as well as in the Victoria Falls region of Zimbawe (18°02′S, 25°45′E). AG332 also spent an 8 day period passing through the Okavango-Moremi protected area in northern Botswana (19°19′S, 22°51′E) *en route* to Namibia. Ivlev's electivity indices were not calculated for AG332 due to its limited tracking period.

### Utilisation of supplementary feeding sites

Excluding AG332 which did not visit a supplementary feeding site after leaving the capture site, the proportion of stationary GPS locations recorded within 1 km of feeding sites for each vulture were 1.81% for AG330, 0.84% for AG331, 26.68% for AG350, 22.72% for AG356 and 9.47% for AG032. The vultures visited between 2 and 4 feeding sites each (mean ± SD = 3.40±0.89), totalling 8 different sites including the MWR capture site. Two of the sites were in the Victoria Falls region of Zimbabwe, and one was south-east of Gaborone in Botswana. The remaining 5 sites were in the North West and Limpopo Provinces of South Africa. MWR was never re-visited by any of the vultures fitted with tracking units after they left the capture site.

Two of the vultures spent a relatively large proportion of their time each month in the vicinity of supplementary feeding sites, with up to 59.15% and 52.22% of stationary GPS locations per month being recorded within 1 km of feeding sites for AG350 and AG356, respectively. AG350 repeatedly spent extended periods at a privately managed supplementary feeding site approximately 16 km south-east of Gaborone, Botswana (24°42′ S, 25°56′E) with 24.05% of all of its stationary GPS locations within 1 km of that site. From April until July AG356 regularly utilised a site approximately 16 km south-west of Victoria Falls, Zimbabwe (18°02′ S, 25°45′E), with 15.42% of its stationary GPS locations recorded within 1 km of that site. The same vulture was also regularly recorded in the vicinity of a second site located several kilometres west of Victoria Falls town at Victoria Falls Safari Lodge (17°54′ S, 25°48′E), where it was seen feeding several times, identified from its patagial tag number. There was a significant negative correlation between the area of monthly path GCRs and the proportion of stationary GPS locations recorded within 1 km of supplementary feeding sites (*r_s_*_(24)_  = −0.674, *p*<0.001, n = 24 months), indicating that the vultures traversed smaller foraging ranges when they were in the vicinity of feeding sites for longer periods.

## Discussion

This study provides the first description of ranging patterns of immature African white-backed vultures tracked from South Africa using GPS tracking technology. The foraging range estimates varied markedly between methods, emphasising the need to use appropriate methods depending on the data available and the aims of the study [27]. As seen previously, MCPs and, to a lesser extent, KDE contours included large areas that were never visited by some of the vultures, especially the widest ranging individuals [28], [38]. Path GCRs reduced the inclusion of unvisited areas and produced the most realistic, but nonetheless conservative, representations of the vultures' movements. The spatial extent of core GCRs and 50% KDE contours corresponded closely and successfully delineated centres of activity. KDE using the *ad hoc* method of bandwidth selection and GCR methods should both be considered suitable for the analysis of similar vulture tracking data.

In general, foraging range size among vertebrates is inversely related to resource abundance and spatio-temporal predictability [39]. The large foraging ranges and relatively long distances travelled by the vultures within their range boundaries indicate that the distribution of their food supply (i.e. ungulate carcasses) was generally unpredictable and sparse, as expected [2], [4]. The maximum distances that the vultures travelled in a single day (mean  = 207.97±17.44 km) confirm that they are capable of searching for carcasses across a vast daily foraging range and that vultures present at a carcass might have arrived from many kilometers away [40]. Although the large overall foraging ranges recorded during this study were expected because immature *Gyps* vultures are thought to move in a nomadic manner from one food source to another [1], [4], the long distance movements from the capture site made by three of the vultures were perhaps surprising in the relatively short tracking periods. These results and re-sightings of marked individuals more than 900 km from their natal origins [19] confirm that immature African white-backed vultures are able to disperse widely across southern Africa, possibly to avoid competing with adults for the same food supply [4].

Although very few foraging range estimates exist for immature African white-backed vultures, and African vultures in general, one study estimated a similarly large mean home range of 482276 km^2^ for two immature Cape vultures (*G. coprotheres*) (one was a possible *G. africanus* x *G. coprotheres* hybrid) based on MCPs from satellite tracking data in Namibia [20]. The foraging range estimates in our study are substantially larger than the estimate of 1940 km^2^ for a breeding colony of Cape vultures in the Western Cape Province of South Africa obtained from landowner questionnaires and radio-tracking data [41], and 9200 km^2^ for a Cape vulture population in the Drakensberg mountains obtained from re-sightings of marked individuals [42]. Comparisons with earlier studies are difficult, however, due to differences in environmental conditions and foraging ecology of the different study species, and the methods used, with continuous GPS tracking methods able to provide a much better representation of the vultures' movement patterns [27]. The foraging range estimates and movements recorded during this study are substantially larger than those from similar GPS tracking studies on *Gyps* species in Asia (mean MCP  = 24155 km^2^ for six *G. benegalensis* [43]) or Europe (median MCP  = 7419 km^2^ for eight *G. fulvus* [44]), and were therefore some of the largest recorded for any *Gyps* vulture species in the world to date. The mean and maximum speeds of travel correspond to early estimates for *Gyps* vultures in the Serengeti [3], [40].

Although Ivlev's electivity index values indicated that three vultures spent more time inside protected areas than expected if they were using protected areas in proportion to their availability, only a small proportion (<5%) of stationary GPS locations were recorded inside protected areas for two of those vultures (AG331 and AG350). The low availability (<4%) of protected areas in the 95% KDE contours of both vultures probably caused the Ivlev's electivity index values to reflect a relatively high degree of positive selection despite use only marginally exceeding availability [34]. The limited amount of time that the vultures spent in South African protected areas indicates that they were able to locate sufficient carcasses to meet their energy requirements by regularly foraging on private farmland. This emphasises that, although the creation of relatively new wildlife reserves such as Pilanesberg National Park and Madikwe Game Reserve in the late twentieth century was expected to benefit vultures in northern South Africa [45], there is an urgent need to implement vulture conservation measures beyond the boundaries of the protected area network. The ungulate populations inside many of the fenced protected areas in northern South Africa are regulated primarily by unusually high rates of predation by large carnivores such as lions (*Panthera leo*) rather than other causes of mortality such as malnutrition [46], [47]. As vultures are known to feed mainly on ungulates that die from causes other than predation and rarely land at carcasses with large carnivores in attendance [1], their limited use of South African protected areas during this study could be partially explained by lower food availability and elevated levels of competition in fenced reserves containing high densities of large mammalian carnivores compared to the much larger protected areas of northern Botswana and Zimbabwe [46].

The geographical distribution of protected areas in northern South Africa might also have reduced their accessiblity to the vultures. For example, several relatively large protected areas within the foraging ranges of the vultures were located in mountainous areas (e.g. the Waterberg Mountains; Pilanesberg) which were avoided by all of the tracked vultures (Fig. 4A). As African white-backed vultures favour flat, lowland savannah [4] it is possible that some of the protected areas in the region are located in areas lacking suitable environmental characteristics (e.g. topography) for efficient foraging activity and are therefore rarely visited.

More than 34% of stationary GPS locations of the two vultures that travelled more widely through southern Africa to northern Botswana and Zimbabwe were recorded inside protected areas, all of which were outside South Africa. Both vultures spent extended periods in the large reserves of the Zambezi-Kavango TFCA, and other reserves outside South Africa, where ungulate densities are higher than surrounding unprotected land and disturbance is comparatively low [48]–[50]. These results support previous suggestions that vultures regularly use protected areas in Botswana and other African countries, probably due to lower levels of anthropogenic disturbance and higher food availability compared to unprotected areas [13]–[15], [51], but also show that the tracked vultures still spent the majority of their tracking periods outside protected areas.

The vultures' core foraging ranges (Fig. 2) were located in areas known to be important for African white-backed vultures, and corresponded closely with high reporting rates for the species recorded during ground surveys [4], [52]. The distribution of ungulate carcasses was probably the most important factor that influenced the movement patterns of the immature vultures because their principal activity would have been searching for food and, unlike adults, they were not restricted to foraging within a certain distance of a nest site [4], [53]. Farming of wild and domestic ungulate species is common and widespread in northern South Africa and southern Botswana, where several of the vultures spent a large proportion of their time [54], [55]. It is likely, therefore, that the vultures consumed carcasses of both wild and domestic ungulate species, as previously seen in the study area and elsewhere in South Africa [56], [57]. The apparent seasonal variation in foraging range size recorded during this study might have been caused by higher mortality rates of wild ungulate species during the dry winter months [58] increasing the ability of the vultures to locate carcasses in smaller foraging ranges. Although mortality rates of domestic livestock are generally higher in the wet summer months [59] their carcasses are more likely to be found and removed by farmers on commercial livestock farms than on more extensively farmed land, such as game farms [4], [56]. The vultures might also be forced to travel further during the wet summer months when increased vegetation causes a reduction in carcass detectability [60]. It was not possible to verify the purpose of the vultures' movements, however, and, as with previous studies that recorded seasonal variations in *Gyps* vulture ranging patterns, the underlying causes remain unclear but merit further investigation [20], [43].

Two vultures were regularly recorded in the vicinity of specific supplementary feeding sites that they repeatedly visited for extended periods, which suggests that they were able to obtain a large proportion of their food requirements at those sites. *Gyps* vultures frequently use supplementary feeding sites elsewhere in southern Africa [17], [20], and the provision of supplementary food at fixed locations has been shown to reduce vulture foraging ranges [43]. Similar patterns were recorded during this study, with smaller monthly foraging ranges recorded during months when the vultures spent a greater proportion of their time in the vicinity of feeding sites. Although not all of the vultures were regularly recorded in the vicinity of feeding sites, it is possible that they visited feeding sites that were not recorded in the database used for this analysis, and so these estimates might be conservative. Further research is required to determine the use of supplementary feeding sites by vultures in southern Africa, and their potential impacts on vulture foraging ecology and conservation [18].

The small sample size (*n* = 6) and relatively short tracking periods (101 – 313 days) limited by financial and technological constraints require that the results be considered with some caution. It was also not logistically feasible to verify the activities of the vultures on the ground or the purposes of their flights due to their frequent long distance movements. However, despite these limitations the regular sampling intervals and high accuracy of the tracking units provide a detailed first insight into patterns of space use by immature African white-backed vultures in southern Africa. For conservation purposes it will be essential to carry out similar investigations into the movement patterns of adult African white-backed vultures as their rates of survival and productivity will determine the persistence of the species into the future [17]. It has been estimated that 50% mortality before maturity would lead to only 4.5% adult replacement and hence adults require a breeding life of at least 22 years to replace themselves [21]. With such long breeding life requirements, even minimal changes to adult mortality, or to the proportion of immatures entering adulthood could result in population declines.

## Conclusions

We have found that immature African white-backed vultures are capable of travelling across the entire region of southern Africa and spend a large proportion of their time outside protected areas. Although based on a small sample size, these findings may have important implications for the conservation of African white-backed vultures. If the ranging patterns recorded during this study are repeated across the wider population, then immature African white-backed vultures have the potential to be be exposed to the full range of threats in southern Africa. Their limited use of protected areas and regular use of private farmland, particularly in South Africa, leaves them susceptible to anthropogenic threats such as poisoning by veterinary NSAIDs or predator control measures. Continuing mass poisonings of vultures in southern Africa therefore pose a serious threat to vulture populations from all countries in the region, and co-ordinated trans-national conservation measures will be required to confront the problem. Our results indicate that monitoring and management of the availability and safety of the food supply outside protected areas will be vital for vulture conservation in southern Africa. The findings from this study also demonstrate that GPS tracking technology can be used effectively to provide detailed information about vulture movements and land use selection, and as a tool to inform the planning of vulture conservation strategies. Similar research is required on adult African white-backed vultures and all other declining vulture species throughout Africa.

## Supporting Information

Figure S1 **Foraging range area curves from incremental area analysis of GPS locations from six immature African white-backed vultures.** The number of GPS locations used to generate MCPs by adding consecutive locations until all locations were used is plotted against the area of each MCP. (A) – (F) represent different vultures.
(TIF)

Figure S2 **Ivlev's electivity index values for protected (PA) and unprotected (Non-PA) areas for five immature African white-backed vultures at the (A) overall and (B) core foraging range scales.** Availability was represented by the relative proportions of protected and unprotected areas in each vulture's 95% KDE contour. At the overall foraging range scale (A) use was represented by the proportion of each vulture's stationary GPS locations recorded inside protected and unprotected areas. At the core foraging range scale (B) use was represented the relative proportions of protected and unprotected areas in each vulture's 50% KDE contours. Ivlev's electivity index values range from −1 to +1, with zero indicating use in proportion to availability, while positive and negative values indicate use more or less than expected, respectively.
(TIF)
