# Supplementary figures and images for: Foraging Ranges of Immature African White-Backed Vultures (Gyps africanus) and Their Use of Protected Areas in Southern Africa
Source: PLoS One. 2013 Jan 30;8(1):e52813. doi: 10.1371/journal.pone.0052813 (PMC3559650; doi:10.1371/journal.pone.0052813)

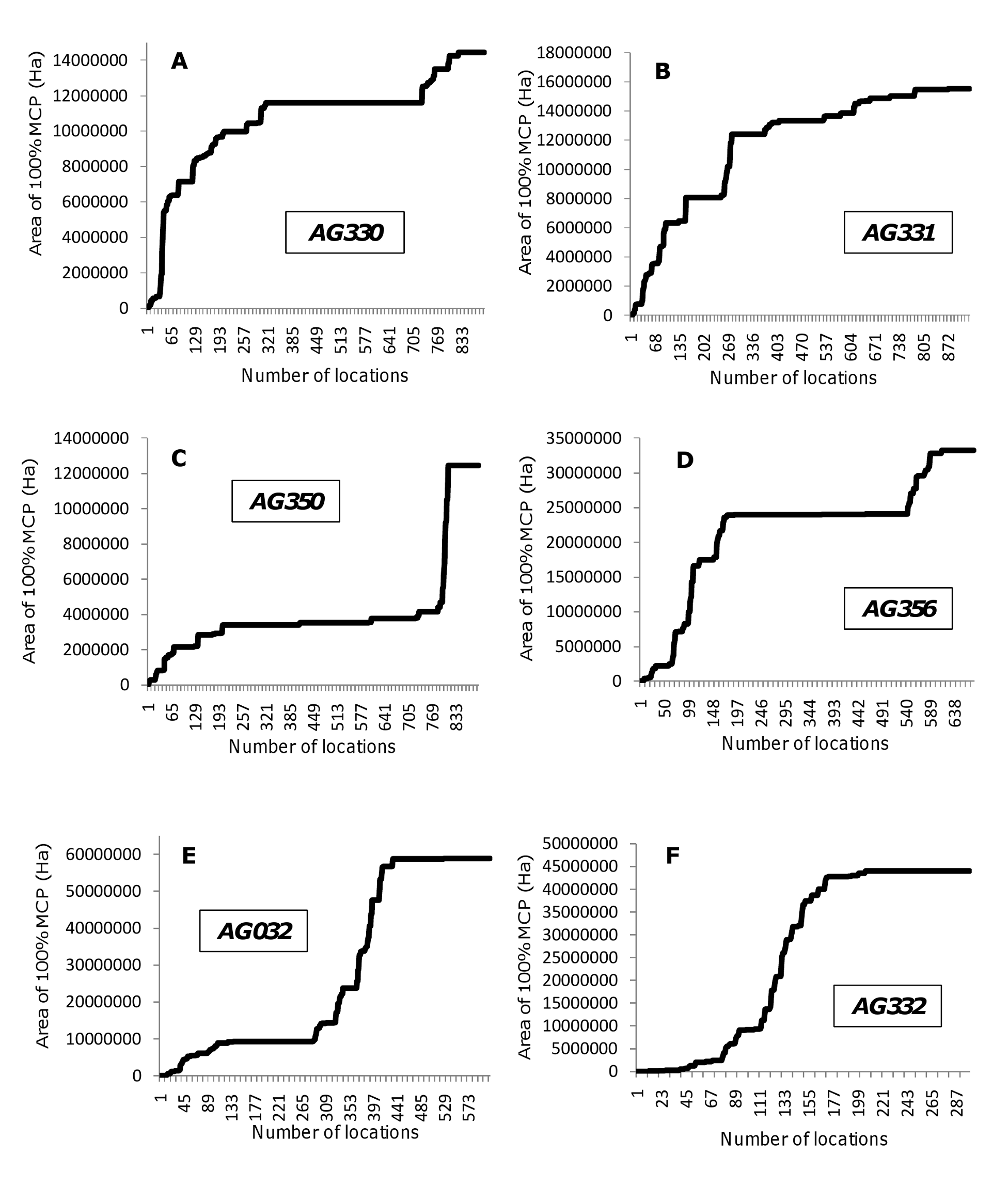

Supplement: Figure S1 — Foraging range area curves from incremental area analysis of GPS locations from six immature African white-backed vultures. The number of GPS locations used to generate MCPs by adding consecutive locations until all locations were used is plotted against the area of each MCP. (A) – (F) represent different vultures. (TIF) [file pone.0052813.s001.tif]

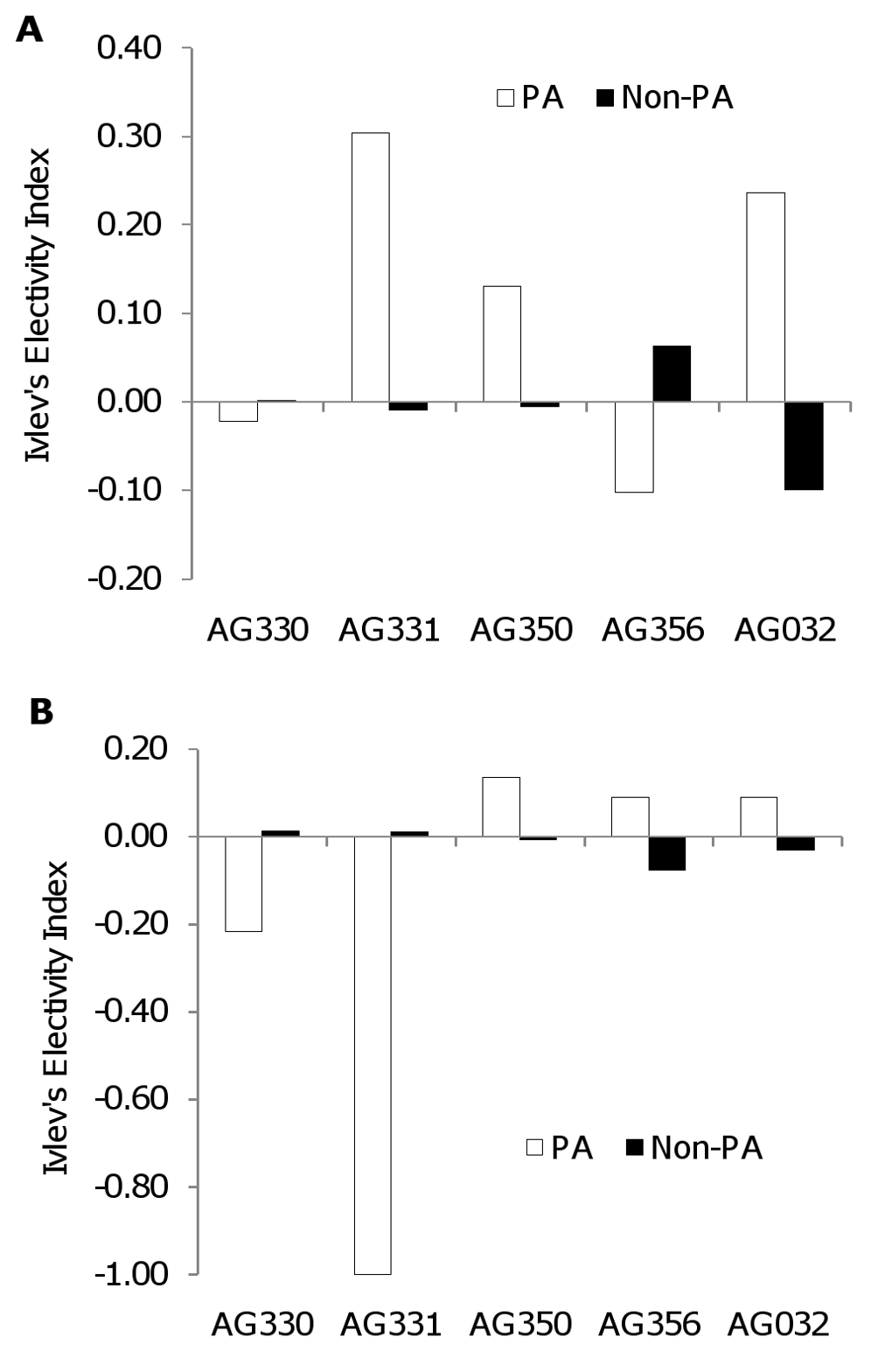

Supplement: Figure S2 — Ivlev's electivity index values for protected (PA) and unprotected (Non-PA) areas for five immature African white-backed vultures at the (A) overall and (B) core foraging range scales. Availability was represented by the relative proportions of protected and unprotected areas in each vulture's 95% KDE contour. At the overall foraging range scale (A) use was represented by the proportion of each vulture's stationary GPS locations recorded inside protected and unprotected areas. At the core foraging range scale (B) use was represented the relative proportions of protected and unprotected areas in each vulture's 50% KDE contours. Ivlev's electivity index values range from −1 to +1, with zero indicating use in proportion to availability, while positive and negative values indicate use more or less than expected, respectively. (TIF) [file pone.0052813.s002.tif]
